# Seeing Cellular Debris, Remembering a Soviet Method

**DOI:** 10.1080/08949468.2016.1131494

**Published:** 2016-02-06

**Authors:** Ann H. Kelly

## Abstract

A 1962 photomicrograph of a mosquito taken in what was then a Tanganyikan mountain laboratory offers a prompt to consider the social salience and affective power of scientific images. Drawing inspiration from anthropological work on photographic practices, this article excavates the diverse geopolitical and domestic contexts of the image's production, consumption and circulation, so as to grasp the relationship between scientific labors and lives. As much souvenir as “epistemic thing,” the photomicrograph provides new directions in thinking about the materiality of memory in tropical medicine.

The photomicrograph acts pedagogically by extending—in fact revising—the process of observation. In short, the photomicrographic trace becomes an archive as a drawing could not; the photograph is a resource for further inquiry.

—Daston and Galison [2007: 178]

Insects are the great progenitors of scientific vision. Histories of the Enlightenment unfold through the painstaking efforts to perceive the barely visible, alien worlds teaming beneath our feet [Daston 2004; Jardine *et al.* 1996; Terrall 2010]. Entomological knowledge was forged from intensive and repeated observations conducted over long periods—a regime of visualization that came to define a new scientific age [Wallmann 2014]. Marvellously strange, insects are also curios, spawning mutant morphologies that confounded the systematics of natural kinds [Daston and Park 2001; Raffles 2010]. Representing nature's exquisite miniatures therefore is not only a matter of magnifying detail, but also of making sense of truly esoteric corporeal forms [Lynch 1985; Meli 2010].

As species of visual pedagogy the sensational engravings that accompany Robert Hooke's *Micrographia* [(1664) 2005] are iconic [Jack 2009]. The “head and eye of the grey drone fly,” for example, portrays the unsettling symmetry of the bristled palps, proboscis and horned antennae—a bearded mouth that verges on the obscene [Figure 1]. It is the eye however that dominates the plate and strains Hooke's analogical imagination. Alternatively a “lattice of cones,” “pyramids” and “golden nails,” the dead eye produces a semiotic surplus, collapsing the subject and object of observation:

**Figure 1:**
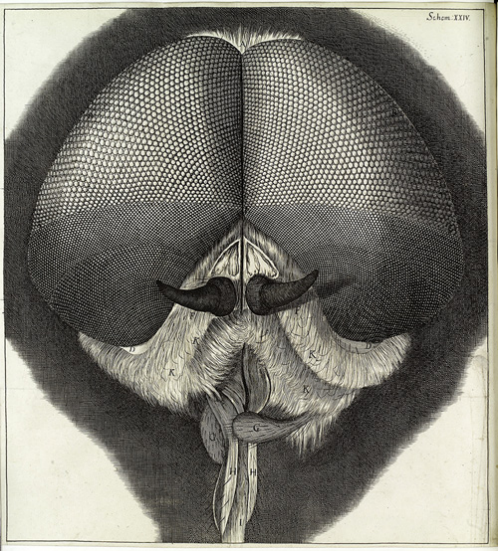
Eye of a gray drone-fly. Scheme 24 of Robert Hooke's *Micrographia* [(1664) 2005]. (© Wellcome Trust. Reproduced by permission of Wellcome Trust. Permission to reuse must be obtained from the rightsholder.)

each of its ‘neer 14,000 hemispheres’ reflecting as exact, regular and perfect an Image of any Object … a Landscape of those things that lay before my window, one thing of which was a large Tree, whose trunk and top I could plainly discover, as I could also the parts of my window, and my hand and fingers, if I held it between the Window and the Object … as they appeared in the bigger magnifying-glass to reflect the Image of the two windows of my chamber [Scheme 23, 24].

The *mise en abyme* of compound eye and compound microscope indexes the epistemic potential of insects, as links between things and processes, particulars and laws, “the surface on which apparatus and objects make contact” [Rheinberger 2010: 217]. But even as the microscope domesticates the insect for the laboratory, the fly's eye wanders into Hooke's bourgeois chambers, reflecting the possessions and private arrangements that provided the setting of experimental science in 17th-century England [Shapin 1988; Campbell 1999: 198–202]. Hooke's microscopic tableau—its play of surfaces and interiors, fingers and hemispheres—unfolds the inner workings of nature within the intimate material assemblage of the domestic space. Scrambling the interpretive conventions of figure–ground, object–context, the plate invites reflection on the image as an expansive arrangement of objects, sites and senses [Strathern 2002].

With that provocation in mind, this article takes as its point of departure another insect image, also produced in a home-laboratory almost three centuries later. The image is a photomicrograph of a dissected mosquito ovary [Figure 2], and was taken in 1962 by T.J. (“Tony”) Wilkes, an entomologist working at the Amani Hill Station in what was then Tanganyika. The ovary belongs to a particularly old mosquito—one that has laid eight batches of eggs. Because female mosquitoes require protein to ovulate, we also know that this specimen has drawn human blood at least eight times.

**Figure 2:**
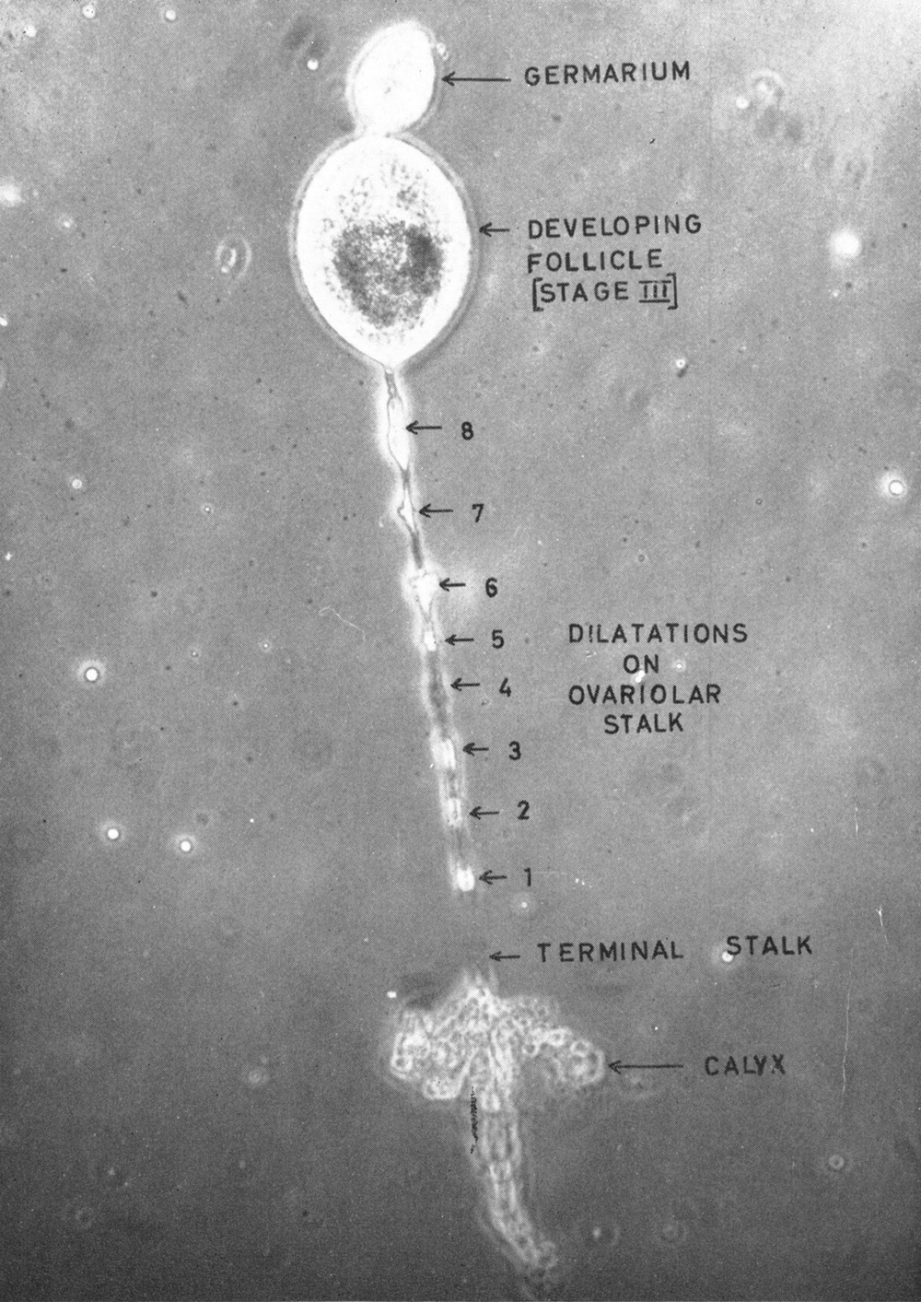
Photomicrograph of an ovariole in *Anopheles gambiae* showing 8 dilations. (Photo from Gillies and Wilkes [1965]; © Cambridge University Press [Bulletin of Entomological Research]. Reproduced by permission of Cambridge University Press [Bulletin of Entomological Research]. Permission to reuse must be obtained from the rightsholder.)

It is a powerful image for those who know how to read it, depicting not only the mosquito's advanced age, but also its epidemiological significance. Because each bite provides an additional chance of the mosquito delivering or acquiring the *Plasmodium* parasite from a human host, the more times the mosquito has ovulated, the more significant its role in malaria transmission. For a short-lived species, this mosquito was a sort of “Typhoid Mary.” Equally impressive is what the photograph reveals about the technical acuity of the dissector. Exposing the scarification within a mosquito's ovary—stringing out the subtle dilations and distensions that an egg will produce as it moves down the stalk—is a procedure few, to this day, have been able to master.

On visiting Tony Wilkes' at his home in Cheddleton, a small town near Stoke-on-Trent, the magnitude of the photomicrograph as a technical achievement becomes clear. Tracing the tell-tale bumps with his finger and then mimicking the gesture of dissection in the air, Tony describes how he managed to draw out the single ovariole from the tightly wrapped cluster—“like a string of pearls”—and lay the specimen on the slide to be photographed. He recalls shaking with nerves in the laboratory's dark room, and how when the image emerged, he ran to his supervisor, Mick Gillies, shouting, “Mick, Mick, look what I've got!” They put their arms around one another and laughed. “It was,” Tony said, “one of the best days of my life.”

Sociologists and historians of science have examined images from a variety of theoretical perspectives, ranging from the ontological status of visual representation to the aesthetic foundations of data analysis [Daston and Galison 2007]. More ethnographically inclined approaches have unraveled the socio-material practices that underpin the production of inscriptions [Kelty and Landecker 2004; Knorr-Cetina 1981; Latour 1986], reflecting on the multiple roles images play in science, as vehicles of revelation or dissemination, rhetorical devices or bulwarks against bias or doubt [Dumit 1999; Rudwick 1976; Tufte 1997]. However, in exploring the specificity of the image as an object of evidence, its subjective affectivities and social capacities can go unnoticed. In Tony's living room, the indexical capacities of the photomicrograph, to either capture natural process or to demonstrate scientific facts, faded from view. Narrated in a nostalgic register, the image provoked an encounter with a lived past, its epistemic immediacy circumscribed by biography.

So if we begin here, with Tony's finger on the print, what kind of trace does the photomicrograph become? How does the image's status as a souvenir complicate its historicity as scientific inscription? This is where visual anthropology can provide conceptual mileage. Ethnographic engagements with photographs and photographic practices have emphasized the multiple agencies of images to extend kinship, enact personhood and transmit cultural memory [Bajorek 2010; Edwards 2009; Empson 2011; Pinney 2011]. In these accounts, the complex temporalities of the photo as past event, present resource and future orientation are negotiated within multiple contexts of use [Buckley 2005]. Attention to the corporeal and material dimensions of those transformations has gone a long way towards opening up the semiotic processes of photographic observation beyond the limited purview of the image's content [Edwards 2012].

Thus rather than contextualize the image *vis-à-vis* the discursive arrangements of scientific practice—e.g., the tools of microscopy or the organization of laboratory work—the analytical task here would involve following the seams that run between the image and the social worlds in which it is experienced [Banks and Vokes 2010]. “Unstitched” from the entomological project, the photomicrograph can, as Daston and Galison suggest, provide “a resource for further inquiry”—though not in this case towards illuminating the natural processes cultured on the slide, but in excavating the intersections of scientific sites, materials and lives, memories and aspirations.

This article develops those connections across three sections, each drawing upon a distinct repertoire of visual materials. I begin with the visit to Amani of Tatjana Sergeevna Detinova, a Soviet entomologist. It was she who taught Tony how to dissect mosquito ovaries and, critically, how to see them. That pedagogical task was hardly straightforward—the arrival of a Soviet entomological method led to a clash of visual cultures amplified by the geopolitics of tropical medicine in the 1950s and 1960s. The article imaginatively extends the inability of Mick Gillies, Amani's senior entomologist, to perceive the entomologically obvious to Communist accusations that the United States waged biological warfare during the Korean War. Against this backdrop, Tony's photomicrograph emerges as a powerful testimony to the existence of a tacit Soviet knowledge and to the potential for a more collaborative global health agenda.

The article then returns to the photomicrograph role in Tony's self-fashioning as a scientist. Here the image is analysed as part of a personal archive—the Wilkes’ family photo album and paintings from their time in Amani. Of particular interest are the domestic reverberations of Tony's entomological work and how his capacities for mosquito perception were situated within the life of the field station. This social biography is offset in the section that follows by the visual traces of local African staff. The strange visual genre of fieldwork portraiture highlights the labor concealed in the photomicrograph and the power dynamics between insect-sight under the microscope and insect-detection in the wild.

The article concludes by following the photomicrograph back into the laboratory during a recent lesson given by Tony at the London School of Hygiene and Tropical Medicine. Watching entomology students take photographs on their iPhones of the ovarial structures Tony displays under the microscope neatly illustrates the multiple historical tenses of scientific visualization and the ways in which insect-vision can come in and out of view.

## MICRO-OPTICS OF SUSPICION

**Figure 3:**
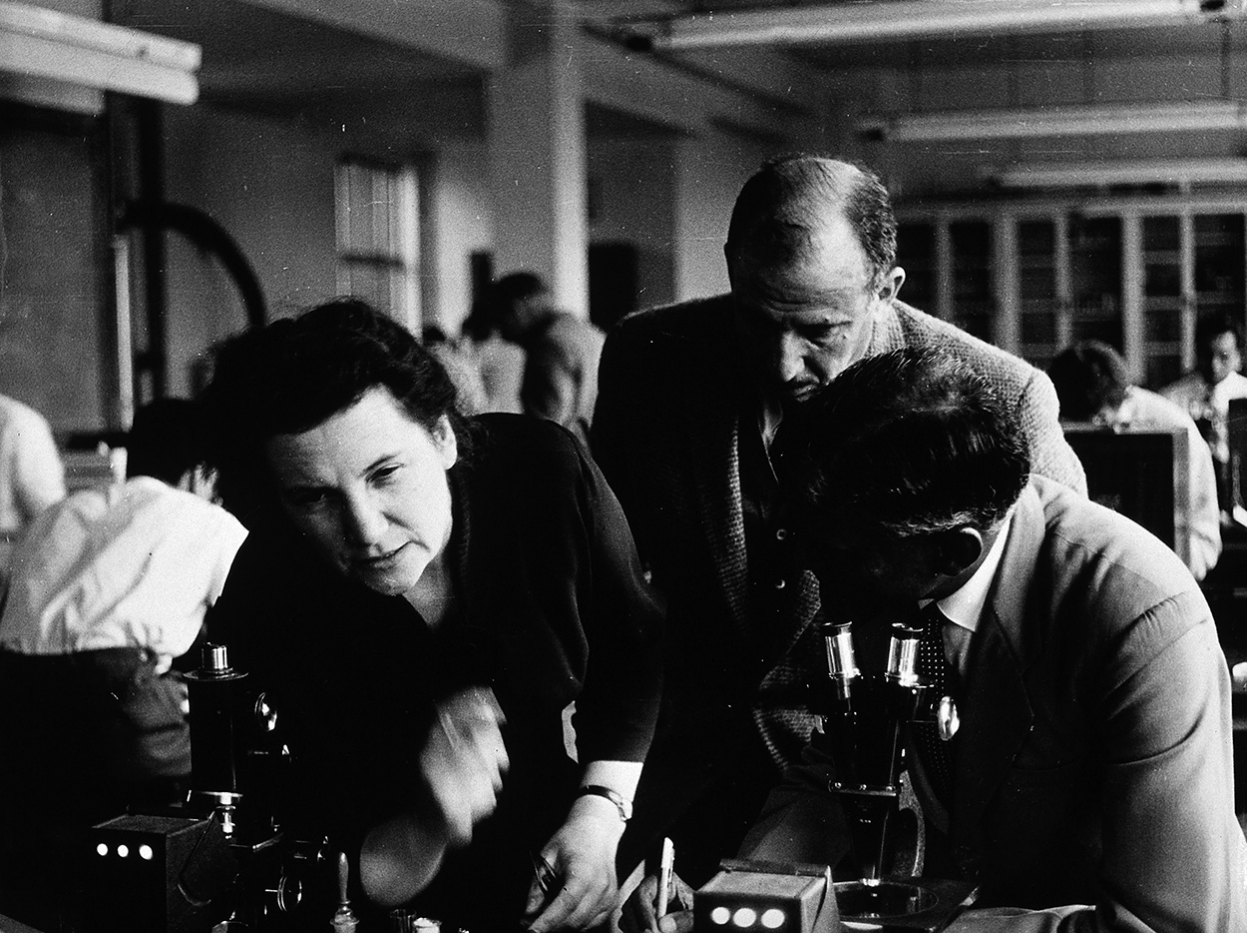
Tatjana S. Detinova teaching at the London School of Hygiene and Tropical Medicine. (Photo by Leonard Jan Bruce-Chwatt, April 1959; © Wellcome Trust. Reproduced by permission of Wellcome Trust. Permission to reuse must be obtained from the rightsholder.)

On September 11, 1962 Tatjana Sergeevna Detinova boarded a flight from Moscow to Cairo to begin a three-month visit to the African continent. An entomologist whose recently translated work was generating considerable excitement in public health circles, Detinova had been commissioned by the World Health Organization (WHO) to run a month-long course at the London School of Hygiene and Tropical Medicine, and subsequently to supervise the application of new entomological methods in the field [Figure 3].

From the Regional Malaria Eradication Training Centre in Cairo, Detinova first flew to Nairobi and then south, on to Tanga, a coastal city in newly independent Tanganyika. Failing to make a connection with her contacts in the East African Commission, and having no Swahili and limited English, she convinced a local driver to take her to Amani—a research station located high in the Usumbara mountains. The driver, who knew about as much about the secluded station as she did, careened the ten miles of hairpin turns up the escarpment into the forest. If it were not for a clerk looking out for a lost vehicle from the cleared slopes of the station, Mick Gillies, Amani's senior medical entomologist, recalled, “the driver would have carried on to the end of the mountains where the land dropped away precipitously and there was nowhere else to go” [2000: 219]. After reaching her destination, Detinova spent six weeks in Amani, before traveling on to field stations in the Republic of Congo, Ghana, Southern Rhodesia, and The Gambia—a three-month-long entomological tour. Despite stomach ulcers, perpetual visa troubles and collaborators of dubious competence, her visit was deemed by the WHO to be a great success.

Detinova's brief was to teach the “Polovodova method,” a technique for establishing the age of a female mosquito which had been developed at the Martsinovsky Institute of Medical Parasitology and Tropical Medicine in Moscow [Polovodova 1941, 1949]. Though she had been refining that method with Valentina P. Polovodova for over a decade, and had published about it in Soviet journals, this work was unknown in the West. During the mid-fifties, when embargoes were gradually lifted, Mick Gillies, who had previously served as the Embassy's Medical Officer in Moscow, managed to locate and translate the missing publications detailing the dissection practice.^1^

In ascertaining a mosquito's age, the method presented a huge improvement on earlier techniques. Existing methods relied largely upon gauging the wear or rubbing on a mosquito's wing scales [Perry 1912]. Ovulation cycles, in contrast, provided a more accurate and relevant basis for assessing age: by factoring the number of eggs against the average number of days required for gestation (2–4 days) and malaria parasite development (10 days), the infectivity of the mosquito could be estimated.^2^ Applied across a large sample, Polovodova's technique could indicate what proportion of mosquitoes were surviving long enough to pose a public health threat, and thus provide a more nuanced model of transmission upon which to base a program of malaria control.

The possibility of gaining new insights into vector longevity is what excited the WHO. At the time of Detinova's visit the Global Malaria Eradication Program (GMEP) had been running at full-tilt for over five years. Its proponents had made a gamble on the residual powers of DDT to kill mosquitoes over extensive periods of time. The theory was that if applied extensively and rapidly enough, DDT could shorten the average life-span of the vector population, effectively preventing the parasite from developing. If in the meantime sick individuals were treated with anti-malarias, mosquitoes would not be able to acquire the parasite from their human hosts and thus transmission could be permanently interrupted within a matter of years [Packard 2007].

In the age of Sputnik, GMEP was to be the great American victory: through sheer man-power, technological prowess, manufacturing efficiency, economic might and optimism, malaria would be eradicated from the world, and Communist sympathies along with it [Spielman and D'Antonio 2001]. Africa complicated that vision: where malaria is holoendemic and the population partially immune, the scalar heroics of malaria eradication lost rhetorical force. For some, the indiscriminate rollout of DDT was a humanitarian commitment; for others, it was potentially catastrophic for African health [Dobson *et al.* 2009]. To moderate that dispute Amani Hill Station became the base of operations for the “Pare-Taveta Scheme,” a large-scale field trial intended to generate precisely the kind of finely grained entomological, parasitological and immunological data that many believed should precede malaria control on the continent.^3^ Insect-sight in Amani was at the crux of defining both the global and the publics of an emerging global public health, and would also test its ideological foundations.

The return of the Soviet Union to the World Health Organization in 1956 amplified those uncertainties. Since the early 1920s the USSR had been engaged in a highly successful malaria eradication campaign in their own country.^4^ In contrast to the GMEP the Soviet approach to disease control was multilateral and community-based, relying on local health networks for intensive disease surveillance and treatment, rural antimalarial stations to coordinate comprehensive environmental management including drainage, filling, insecticide application and a long tradition of entomological and ecological expertise connected to regional stations for pest control and forestry experimentation [Loskutova and Fedotova 2015].

Outspoken against vertical public health interventions of any kind, the Soviet delegation to the WHO played into the rising doubts on the feasibility of mass-spray campaigns [Litsios 2002]. The translation of Detinova and Polovodova's work came at an opportune moment, providing a resource for a critical reassessment of the global eradication agenda. Vladimir Nikolaevich Beklemishev, the Head of the Medical Entomology Department at the Martsinovsky Institute in Moscow (the heart and soul of USSR anti-malarial control) put the potential impact of the technique in no uncertain terms:

As far as malaria eradication is concerned, it is hoped that the methods described in this monograph will serve as a starting-point in all countries for an extensive study of the age composition of mosquitoes in connexion with the epidemiology of this disease. I am convinced that international co-operation in this sphere will lead to the further development of the study of vector biology and thus to the speeding-up of global malaria eradication. [WHO 1962: 11]

Beklemishev, who spent his career illuminating the unknown features of invertebrate bionomics and biology, advanced a malaria control agenda that was grounded in an understanding of the vector's “life-scheme”—or rather the total set of possible interactions between a specific mosquito population and its ecosystem [Beklemishev 1969]. This conviction of the public health powers of close insect surveillance was one that his pupil, Detinova, shared. It fueled her visit to the London School of Hygiene and Tropical Medicine in the spring of 1959 and carried her aboard her Cairo-bound plane.

Not all of course were convinced of the technique's applied potential. For one thing, the Polovodova method proved almost impossible to learn. Among the 15 international entomologists who had been invited to Detinova's course in London, the few who could actually perform the dissection were not in the least confident of their assessments of their specimens’ age [Corradetti 2014]. Mick Gillies, who was responsible for bringing the technique to the West, found himself utterly defeated by its technical subtly. Incapable of making sense of the pigmented bits of ovarial tissue under his microscope in Amani, he began to wonder whether the method could even be applied to smaller African mosquito species. In a report summarizing the outcomes from Detinova's course, Gillies conceded: “Polovodova's full technique was a research tool and normally has no place in the routine assessment of control measures in the Tropics” [WHO 1960]. His opinion was widely shared.

In the hope of restoring faith in the technique's applicability and at the urging of the Soviet delegation, the WHO sponsored a further series of Detinova's on-site lessons. This pedagogic mission brought her into contact with an unfamiliar sociopolitical and ecological landscape, traversed cheek-to-cheek with her students in the intimate spaces of the stereomicroscope. In his autobiography, *Mayfly on a Stream of Time*, Gillies describes the spectacular distances at play in these moments of optic proximity: “She would give me one ovary and look at the other herself. I would then be asked how old I thought it was and have to make the embarrassing reply, ‘*ne znayu*—I don't know.’ I could sense the beginnings of a difficult political situation. I saw myself being cast in the role of a Western scientist refusing to accept a Soviet discovery. Why else couldn't I see what was so obvious to her?” [2000: 222].

While the post-Stalin era saw a rise in “science diplomacy” fundamental differences in the organization, orientation and ethos of research stripped away the trust upon which the circulation of facts and scientific insights rely [Graham 1966; Ivanov 2002]. Coinciding with the Cuban Missile Crisis (1960), Detinova's visit certainly did not escape what Gillies refers to elsewhere as the “shadows of the Iron Curtain” [2000: 224].^5^ This moment of entomological uncertainty however had a specific antecedent in the Korean War. In the spring of 1952 the United States was accused by the People's Republic of China of launching a mass germ-warfare campaign in Gannan County, along the border between North Korea and Manchuria. According to reports, U.S. aircraft had made approximately 1000 passes over the territory over a period of two months, dumping pathogenic payloads of malaria-infected mosquitoes, plague-ridden fleas, anthrax-laced spiders, typhoid-carrying tarantulas, cholera- and dysentery-coated flies and a host of other poisonous and destructive species [Lockwood 2009].

After the Chinese refused to cooperate with the World Health Organization and the International Red Cross, which they believed (correctly) were biased in favor of U.S. interests, the Soviet Union set up an International Scientific Commission to look into the allegations. Joseph Needham (1900–95), a prominent British biochemist who had spent many years in China, was appointed to act as chair.^6^ While none of its members carried out field investigations the mission collected microscopic slides, public health records, autopsy reports, and testimonials from American pilots, Chinese bacteriologists, entomologists, botanists, technicians and villagers from across the benighted region. Indeed it was the intense degree of expert and lay entomological scrutiny that gave the Chinese accusations weight: the detailed descriptions of bizarre species, the close-up photographs of antennae and wings, and magnified representations of bacteria that graced the pages of newspapers seemed to surpass the machinations of a high-level government subterfuge.^7^ While enough to convince the Commission this visual record did not satisfy the United States, who dismissed it as suspiciously extravagant and scientifically thin, failing to draw any conclusive connections between photomicrographs of bacteria and the alien vector species found in Gannan. The WHO and the international community rallied around the United States, who came to regard these entomological anomalies as a confluence of the environmental disturbance occasioned by war and an unseasonably warm spring [Rogaski 2002].

I mention this episode in Cold War science fact-and-fiction not to make sense of the discrepancies between what Gillies and Detinova saw but rather to flag the political salience of entomological vision at this particular moment in time. The Soviet–U.S. dispute over whether or not insects were seen and, critically, what seeing them meant, draws together a number of threads in the reception of Detinova's method. First, there is the ontological status of the entomological trace; or what public health or wartime realities are called into being by an antenna or an ovarial stalk. Secondly, there is the popular character of insect-sight and the salience of locally embedded vigilance in revealing environmental change. Finally—and this goes to the root of Gillies’ anxieties—is entomology's epistemological ground; the methods and material assemblages through which scientists recognize and corroborate the phenomena they study.

In contrast to the findings of the Soviet-sponsored commission Detinova's assessments of mosquito age in Amani's laboratory were validated—though not by Gillies, the great master, but by his assistant, Tony Wilkes. Wilkes was, by all accounts, a “natural”: in possession not only of the necessary dexterity to perform the dissection but also of the visual acuity to distinguish ovariole dilations from shadows of frayed and shrunken cellular debris. While Gillies would go to great lengths to confirm the accuracy of Wilkes’ assessments (including running blind tests using laboratory-reared mosquitoes) the eight-“bump” photomicrograph testifies to Wilkes’ ability, both to recognize the subtle changes in the mosquito and to render those vital signs visible to others.

Gillies, for his part, never managed to come to grips with the method—a shortcoming that he made sense of in terms of a lack of visual imagination:

the main difference seemed to lie in our respective abilities to build up a composite picture of the conditions of the ovaries from fleeting glimpses of a series of ovariolar stalks, each of which might only be fully extended for a matter of seconds … It thus appears possible that the difficulties encountered by the senior author, and perhaps by some other workers, may have been due to an ingrained tendency to be too systematic, to assess each ovariole in isolation from all the others in an attempt to be ‘objective.’ [Gillies and Wilkes 1965: 239]

Stifled by a “too rigidly disciplined mind,” Gillies came to rely on Wilkes for studies into mosquito survival [*ibid*.: 204]. After eighteen months of dissecting and age-grading of mosquitoes in Amani, their work demonstrated that a significant reservoir of infected mosquitoes had indeed survived the intensive spraying of the Pare-Taveta scheme. “It was not,” writes Gillies, “an encouraging discovery” [2000: 225]. By the time Gillies and Wilkes had published their results however the global health community had already backed away from the eradication agenda, first in Africa and gradually everywhere else. According to G.A. Novgorodcev, a Soviet delegate to the WHO, the failure to interrupt transmission was predictable: “the methods and principles adopted for malaria eradication had been over-simplified and were too much alike for all countries” [WHO 1966: 246].^8^ As calls to down-shift malaria eradication to country-led programs intensified, entomological surveillance took a backseat to the promotion of basic health services, prompt diagnosis and treatment [Litsios 2000]. Finely grained insight into the life-scheme of mosquitoes had only proved their elusiveness as a target of control.

At the very least however Detinova's efforts at visual enculturation had not been in vain. Nowhere on her travels would she find a pupil as gifted as Tony; he remains today one of the few emissaries of this style of Soviet entomological sight. Gillies sums up this triumph of technical translation with characteristic wryness: “in Tony, she was leaving behind an enthusiastic recruit capable of carrying out her work and knowing that he would get wholehearted support—or was it exploitation?—from his boss” [2000: 223]. That relationship between Wilkes and Gillies, which would define both men's careers, was cultivated by the sociality of the Amani field station, which integrated scientific work and colonial life. Above the valleys and in the clouds, the lone entomologist at the microscope is just one in a series of insect-visions.

## COLLECTION VIEWS AND VISTAS

In the Wilkes’ living room in Cheedleton Tony elaborates the anecdote of his first ovariole dissection. It begins with a bet: Mick would let Tony try the technique if the latter promised to buy the former a beer for every specimen he got wrong. It was Mick who ended up buying—and as Tony tells it—enough rounds to make him fairly tipsy. A classic Wilkesian anecdote, “polovodova-over-pints” neatly captures the affability and breezy bravado characteristic of “Uncle Tony” (as his colleagues know him). It also hits the various notes of gender and class that structured Tony's life and work at Amani.

When asked to reflect on his scientific career Tony dispenses with any sense of vocation by cataloguing a chain of serendipitous events. After he was kicked out of school in Potter's Bar for skipping class to play football, he joined the army. When it was discovered Tony had lied about his age he was kept out of active duty, spending his three years of service during the Korean War based at a garrison in Hong Kong. It was there where he learned “about bugs, and how hard it was to get away from them.” Unsure of what he wanted to do when he returned to civilian life, Tony answered an advertisement for an entomologist in the newspaper. He was hired on the spot. Bagster-Wilson, Amani's director, was a former lieutenant colonel and, according to Tony, strongly sympathetic to army men.

As to his aptitude with the microscope, Tony defers much to Mick: “When you are working with someone who is *good* and you are working with them for years, it rubs off on you, you don't have to be clever, you just go along with it.” His deference however goes beyond that of a student to his mentor. Tony prized their friendship, a camaraderie that crossed class boundaries and extended over many years. Mick grew up in London near Hampstead Heath, attending the exclusive Winchester College and then going on to Cambridge. His father, Sir Harold Gillies, was a renowned plastic surgeon who had invented an instrument of skin transplantation that became critical for war victims. Following military service in the Far East and a year at the British Embassy in Moscow, Mick received a doctorate from the London School of Hygiene and Tropical Medicine, and took up the post of the senior medical entomologist in the newly formed East African High Commission Medical Research Institute at Amani.

They must have formed a formidable double act: Tony warm, enthusiastic and physical, Gillies quiet, fastidious, with a bone-dry sense of humor. The differences in their respective backgrounds provided plenty of material for practical jokes: switching nametags at conferences was a particular favorite, much to the dismay of star-stuck students. “The look on their faces” Tony recalls, “when they realized they had confused the Genius for the Oaf was priceless.” When they traveled Tony liked to tease Mick by reading aloud “facts” from the *Reader's Digest*.

It is not difficult to imagine how such a relationship could form in a tiny ex-pat community on top of a mountain. With its own hydroelectric dam and generator, dairy herd, post office, church and social clubs, these “scientists in the clouds” (as they were called by researchers working in the valleys below) could pursue their empirical passions disentangled from the teeming social, pathological and climatological realities of the Tropics. “It would be hard,” Gillies writes, “to discover a more secure retreat from the tensions of a troubled world” [2000: 130]. For those residents excluded from its scientific mandate, however, life at 3,000 ft. above sea level was experienced rather differently. Amani's utopic vision—“to carry out laboratory work of precision and to do a prolonged day's work without undue fatigue to the European”—cemented the station's coherence as a home [Bagster-Wilson 1952: 20]. The work of grafting a domestic existence onto a mountain laboratory was borne by the wives of the scientists, who often struggled to find their place within the institutional landscape.

As Tony retreats to his study to find an original print of his eight-bump photomicrograph, Dorothy, his wife, opens up the family photo-album. There are pictures of her garden and baobab tree, children in swimming suits and women in sun-hats having drinks by a lake (“we made our gin and tonics strong”). Occasionally Tony appears in a shot—a family safari or his son's birthday—but he is largely absent. Pausing on a scenic photo of a village at sunset Dorothy explains: “Tony is probably off somewhere collecting mosquitoes behind those huts.”

**Figure 4:**
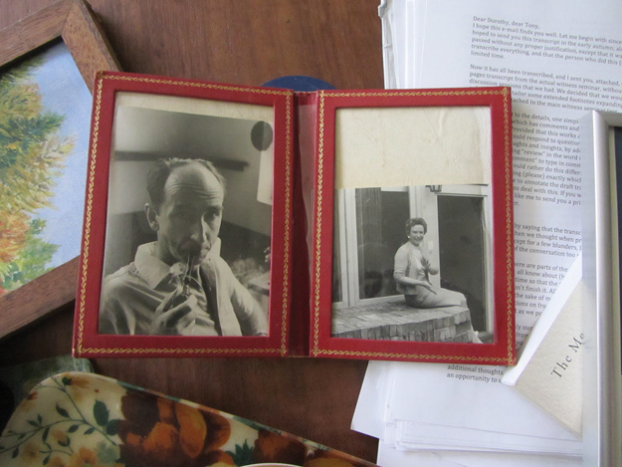
Mick and Aggie Gillies (ca. 1961), framed by Dorothy Wilkes. (Photo © the author, 2014)

Fundamentally women's work, the display of family photos cultivates togetherness. As Gillian Rose argues, the simple act of looking through an album can materialize absent relations and “impart efficacy at turning house into home” [Rose 2003: 10]. Dorothy retrieves a double-picture frame from the mantelpiece: on the left, Mick is seen with his pipe in his laboratory office, a formal portrait that I had come across in the archives at Amani. On the right is his first wife Aggie, smiling on her porch in Sussex. Placed side-by-side, these two images articulate not only the relationship between husband and wife, but also their individual connections to Tony and Dorothy and to Amani—integrating an experience of kinship across place and time [Figure 4].

Mick and Tony met as colleagues and their friendship flourished through their work. Mishaps in the laboratory and adventures in the field—the lion that chased Tony from a collection site—are the leitmotifs of the anecdotes that Tony tells about Mick and that Mick writes about Tony. Amani offered a perfect backdrop for a familiar genre of research expedition: a life of scientific exploration, sacrifice and survival, “a naturalist's dream” [Gillies 2000: 132].

As Tony and Mick made their daily journeys to collect mosquitoes in the valleys below, Dorothy and Aggie's friendship was confined to the mountaintop. For Aggie, who had been living in Amani for almost a decade before the Wilkes arrived, it was indeed a confinement. Aggie had trained as a surgeon, one of few women surgeons at that time. When she traveled with Mick to Amani in 1950, she had left behind a registrar's position at an orthopaedic hospital in North London. She had two daughters in Amani and her eldest, Susie, remembers her mother's profound regret at having abandoned her career: “She felt she had no purpose. She taught classes to African women on how to sew patches on clothes. It was awkward for all involved.” During the rainy season, which lasted many months, the house was cold and dark, constantly under siege from snakes and biting insects. When Mick took up a position at the British Museum, Aggie was ecstatic. Susie describes the return to England as her mother's halcyon days, utterly transforming her mood. Indeed, in the shot on the right, she appears to be clapping.

**Figure 5:**
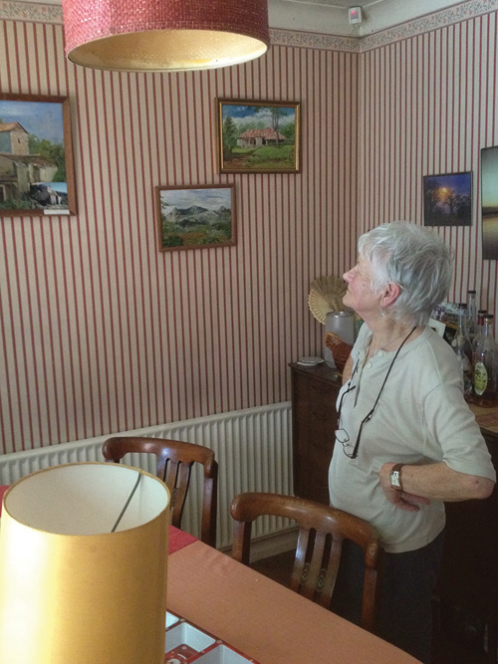
Dorothy Wilkes and her oil paintings of Amani. (Photo © the author, 2014)

In Amani it was painting that had saved her. During the long hours alone while her girls boarded at a school in the valley, Aggie would carry her easel around the station grounds searching for views to render in oils. She had talent: as Jackie, her younger daughter put it, “a voice all her own.” One of her paintings even found its way into a Royal Academy exhibition: “a spare but evocative picture of a tropical backyard, just a tap and the palm-like shadows of a half-seen pawpaw tree draped over the outside wall of the kitchen” [*ibid.*: 199]. Following Aggie's lead, Dorothy also took up painting. While she had sketched on occasion, it was not until she had enrolled in art classes with Aggie during holidays in Sussex that she began to paint with any conviction [Figure 5].

Aggie's artistic potential was tragically cut short. While driving to a Christmas party back in Sussex the two women were hit by a car coming out of a blind drive. The collision killed Aggie immediately and left Dorothy in a coma for a week, with broken legs and ribs. Upon her return to Amani Dorothy continued to paint and, like Aggie, her compositions captured station views and village vistas from the occasional trip to town. A play of blue skies and cloud cover, palm trees and red roads, the paintings articulate the absence at the heart of the hill station's domestic experience: “it was something to do while Tony was away in the labs.”

What do Dorothy's renderings of entomology's adjacent landscapes add to our understanding of Tony's photomicrograph? In her analysis of the aesthetics of British hill stations in India (there are over 80 of them) Jill Dildur suggests that by filtering out any traces of either local industry or colonial intervention, the picturesque gaze naturalized imperial presence and power. The gardens and architecture of remote stations and the techniques of travel writing and painting taken up by their residents reframed an unruly wildness within a familiar perspective. The visual taming of the landscape also reinforced the social and political distance cultivated by colonial women. The picturesque gaze, Dildur argues, acted as a way to “manage their ‘self-image’ as mere spectators (rather than perpetrators) of the violence and exploitation of empire” [2013: 508; Figure 6 here].

**Figure 6:**
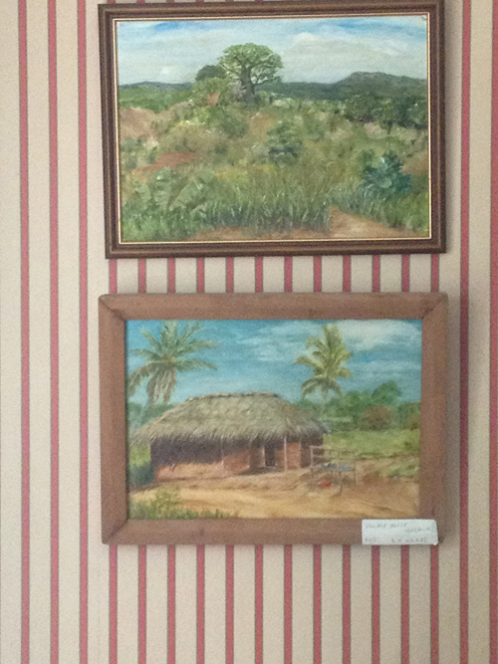
Dorothy Wilkes’ paintings of Muheza, 1987. (Photo © the author, 2014)

Though her commentary explores the sociopolitical dynamics attendant to the final days of the Raj, Dildur's reading of the hill station's conjugation of romance and domination, immersion and detachment resonate with the aesthetics and scientific priorities at Amani. While fundamental to its notoriety as a research site the picturesque ultimately precipitated the station's decline in the years following Tanganyika's Independence. In late 1962, just days after Detinova concluded her African tour, Nyerere was installed as president. Celebrations (largely segregated) were held at Amani; Aggie baked an *Uhuru* (freedom) cake.^9^ Initially the routines of their scientific work acted as a bulwark against these political transformations; research into vector biology continued apace. But as the colonial administration was gradually appropriated by the new authorities, the orientation and organization of scientific work at the station shifted. European scientists accepted generous severance packages—“a golden handshake”—to ease their departure from positions now retained for Africans. Amani's appeal as a scientific retreat began to fade. The twenty miles of hairpin turns that shielded the station from the “troubles of the world” also cut it off from social life, good schools and economic opportunities. For an ambitious Tanzanian cohort the station felt less like a sanctuary and more like a penitentiary [Poleykett and Mangesho, forthcoming].

When Tony and Dorothy returned to the station in the late 1980s they found it much changed. The dissolution of the East African Community following the Tanzania-Ugandan War and the deepening economic crisis severely hampered the state's capacity to fund scientific research. The work that continued was supported through overseas collaborations, and easier to maintain through the better-connected lowland station in Muheza. Amani's scientific program shifted from cutting-edge malariology to studies of traditional medicine. “It was still the same old Amani,” Tony conceded, “the forest, the views. The houses had changed a little. But the place was neglected: it was dying.” After all that work (the discovery of four new species of mosquitoes!) Tony found the empty laboratories, the people just sitting around, “embarrassing.” Dorothy experienced a similar sense of loss. More attuned to the station's physical layout, she was surprised to find that the gardens were gone, the lawns overgrown, and the tennis courts demolished. She had plans to spend her time painting, but struggled to locate familiar Amani panoramas of the green mountain peaks falling towards the sea. “Because the *trees* had grown so much, so high, the views were lost.”

The aesthetic vision of the station—its winding forest paths, cool climate and tree-shaded lawns—spoke to a European, or more so British, cultural imaginary [Geissler and Kelly, forthcoming]. The same might be said of the scientific research, which, while advancing the state of entomological science, remained removed from the immediate public health concerns of the new Tanzanian nation. Tony and Mick had undertaken their age-grading analysis more than three years after the Pare-Taveta experiment had come to an end, and when large-scale spraying in Africa had been abandoned in favor of the administration of anti-malarial drugs.^10^ Amani's remit was to generate basic facts about transmission dynamics that were of global importance; their applications to malaria control and urban engineering were left to the technical departments of lowland stations. Like a picturesque painting, hilltop colonial science cultivated distance from the particulars of place and its inhabitants. Insect-sight of the expatriate kind, domesticated the landscape, rendering it knowable by placing it into the small compass of picture frame or microscope slide.

## VISUAL WORK

A final set of visual materials restores the background detail of entomological sight and sites obscured by the photomicrograph. In the paper where Wilkes’ eight-bump dissection appeared, the methods section elaborates what is involved in analysing the age-structure of a mosquito population:

Mosquitoes were collected by spray-catching with pyrethrum in houses. At Muheza, the catches were made in six small villages, all within 1.5 miles of each other, each usually being visited once every two weeks. A total of some 20–30 houses was collected in during each round of catches … it is considered that the catches, which yielded over 10,000 females, were sufficient to give a good general picture of population trends throughout the year. [1965: 239]

Vector sampling is labor intensive and time-consuming; mosquito catchers slowly comb the surface of a hut, looking for movement along the thatch eaves and under tables, tapping hanging clothes, curtains and kitchenware. When spotted, mosquitoes are sprayed with insecticide or sucked into aspirators. The samples are placed in damp cloth-lined tins, counted and classified into male and female, blood-fed or pregnant—a process that requires meticulous attention and delicate fingers. Following scientific convention, these labors are written out of Gillies and Wilkes’ account by the passive voice.

**Figure 7:**
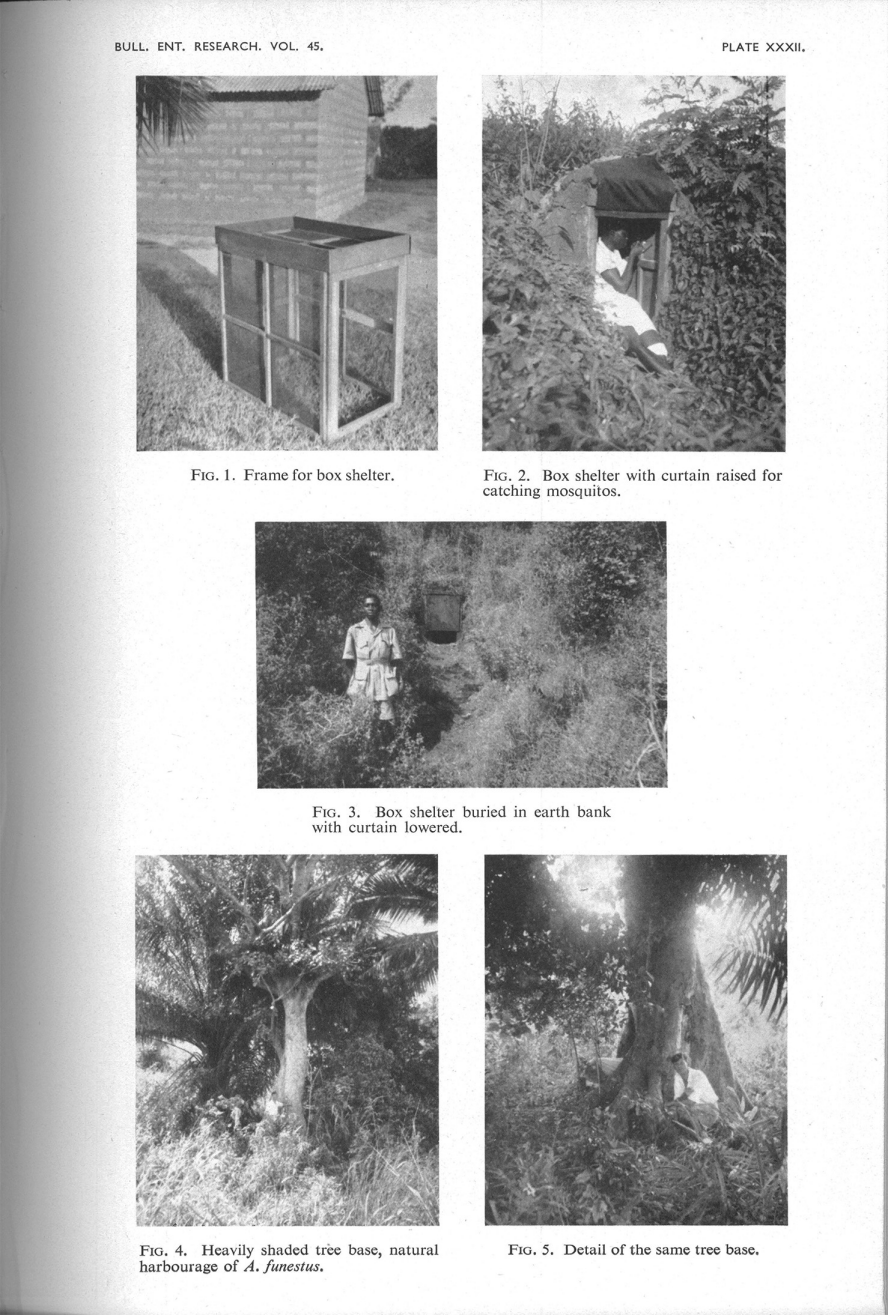
Images showing artificial shelters to collect mosquitoes from outdoor resting sites. (Photo from Gillies [1954]; © Cambridge University Press [Bulletin of Entomological Research]. Reproduced by permission of Cambridge University Press [Bulletin of Entomological Research]. Permission to reuse must be obtained from the rightsholder.)

It is precisely in the descriptions of method, however, where this work tends to reappear. Pictured within a natural setting, mosquito catchers are critical elements in the visual vernacular of entomological research. The strange gallery of trapping boxes, suction tubes, nettings and wind-tunnels presented in the final plates of scientific papers are inevitably accompanied by mosquito catchers (or, by the mid-1960s, “technicians”) collecting, counting, spraying or sometimes simply standing to provide a sense of scale. Like Bronislaw Malinowski's famous “ethnographer's tent on the beach of Nu'agasi” [1922: 6, pl. 1], these images stage the “field” in a manner that reinforces the representational naturalism upon which field research is based. The catchers also act as the scientist's proxy: confirming his “having been there” while not personalizing his objective purview [Figure 7].

The view of reality revealed by these photos is clearly not of the same order as an ethnographic print. What is presented is not the empirical subject—in this case, mosquito bodies, behaviors, or even (at least directly) their habitats. Rather it is the technical ingenuity of experimental design that is on display—a visual record of entomological pragmatics. Like the forest wood used to make the “box shelter,” the mosquito catchers articulate an aesthetic and moral commitment to the makeshift constructions, simple methods and materials-to-hand [Kelly 2012]. They are figments in a naturalist's dream—part tropics and part experimental infrastructure.

**Figure 8:**
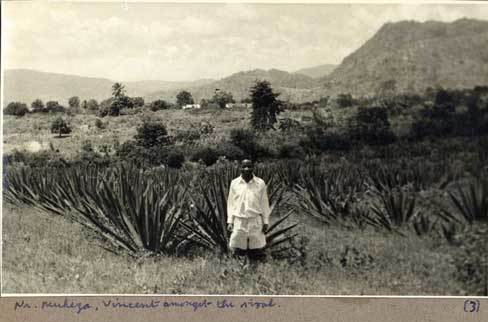
Nr. Muheza, Vincent amongst the sisal. (Photo © Chris Draper Archive, London School of Hygiene and Tropical Medicine, 1959. Reproduced by permission of Chris Draper Archive, London School of Hygiene and Tropical Medicine. Permission to reuse must be obtained from the rightsholder.)

Deeper into the entomological archive the representational status of the mosquito catcher becomes more ambiguous. Chris Draper, who oversaw the Pare-Taveta scheme, kept an extensive photo archive of his time working in the lowland areas around Amani. The pictures were taken during collections and feature one of his three technicians—Vincent, Samuel or Omari. Some look like outtakes from the methods section of a scientific publication: Omari, for instance, standing by a hut, spray canister in hand, with the inscription: “catching anophelese at Mkomazi.” Others are more candid: a shot of Samuel standing beside an open car door reads “Muheza: a base for reconnaissance”; another, reminiscent of a souvenir from an East African safari, reads “Near Muheza, Vincent amongst the sisal” [Figure 8].

These images, like Dorothy's and Aggie's paintings, belong to the picturesque; tinged with sentimentality, their composition—a smiling figure amid the flora, rolling hills in the background—interiorizes exotic space, creating distance from lived experience. The relationship between Vincent and Draper however stretches those conventions: neither family member nor charming native, Vincent's presence reframes the landscape as taskscape [Ingold 2000; Scherer 1990]. Dressed in his uniform, looking directly at the camera, he indexes not merely the event of “being in” a place but also memorializes the efforts to improve it. Again he stands-in for the scientist-photographer, justifying his authority to not merely document but to intervene.

**Figure 9:**
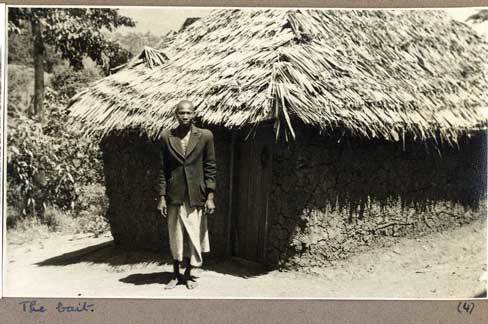
The Bait. (Photo © Chris Draper Archive, London School of Hygiene and Tropical Medicine, 1959. Reproduced by permission of Chris Draper Archive, London School of Hygiene and Tropical Medicine. Permission to reuse must be obtained from the rightsholder.)

A final image exemplifes those power-dynamics. The shot itself is unremarkable: the figure in the foreground, barefoot, dressed in a jacket and kanga, stands in front of what presumably is his hut. What transforms the scene is the caption, which simply reads: “The bait” [Figure 9].

To elucidate this somewhat macabre entry into the archive, some entomological background is necessary. The most effective and accurate way of sampling vectors is to catch the mosquitoes as they come to feed. Human-baiting remains the gold standard in assessing the rates of malaria transmission, but requires a human subject to act as lure. This tedious task would often fall to technicians who would sit for hours, generally at night, waiting for mosquitoes to land on their exposed legs. Another method would involve taking local volunteers who would be asked to sleep under nets or in experimental huts, from which technicians would gather mosquitoes in the morning. That methodological context however does not exhaust the semantics of this annotated shot. On one hand, there is a cynicism to the label's bluntness—a gallows humor that both acknowledges and shrugs off the violence of imperial science. On the other, the posing of the nameless, unsmiling African next to his village hut links this image to an emerging genre of humanitarian photography [Heide and Rodogno 2015]. While not visibly suffering, the figure is inscribed as part of a population afflicted by malaria. In pathologizing the subject, the photo also annunciates its project of salvation: to protect these people from their fate as unmonitored, collective bait.

Images of labor anchored the moral value of entomological work as both scientific endeavor and colonial development. They articulate the naturalism of the field and the resourcefulness of the scientist. Amongst the sisal and against the escarpments, they anticipate the memory of a global health mission. Their situated insect-vision, often effaced from evidentiary claims, provides the connection between scientific work, place and meaning.

## CODA: POINTING TO THE PAST

This article began with Tony's finger on a photomicrograph. The gesture embodies science in action; “the extension of the finger,” Latour argues, “always signals an access to reality even when it targets a mere piece of paper” [Latour 1999: 65]. Pointing orients empirical attention, defines a field of inquiry, and aligns representation with scientific discourse. The finger, quite literally an index, sets off a series of transformations between matter, image and text that gradually release facts from particular places and moments in time.

The reality signaled in the Wilkes’ living room in Cheddleton lies outside the circulation of scientific references. Tony's finger on the photomicrograph refers back not to the age of the mosquito, nor to the number of infective bites it has delivered, but rather to the memory of a scientific life. In this article I have sought to unravel that past through a series of visual translations—from lab. to home to field—that re-contextualize the image against different insect-visions at work in Amani. So what might that analytical backdrop illuminate about the photomicrograph's salience today?

Let us consider one final image and index of the Detinova method. Though long since retired, Tony Wilkes will occasionally make the trip down from Stoke to lecture at the London School of Hygiene and Tropical Medicine. Mark Rowland, an entomologist who has also worked for a number of years in Tanzania, introduces the lesson: its central message is the continuing importance of mosquito longevity to the success of malaria interventions. Tony's photomicrograph made the point: the age and not the density of mosquito populations is what matters for transmission [Roth and Bowen 2001]. As insecticide resistance gains a foothold in sub-Saharan Africa, the method has taken on a renewed importance: there are African teams based in Benin and Tanzania who are working again with the method; some using new 3-D technologies to better capture the ovariolar stalks [Anagonou 2015; Hugo *et al.* 2008; Mayagaya 2015]. Beyond the technical means of visualization, this research requires an extensive network of rural surveillance—or what Raphael N'Gessun, a scientist in Contonou, describes (evoking the Soviet model of malaria control) as “an entomologist in every village.”

**Figure 10:**
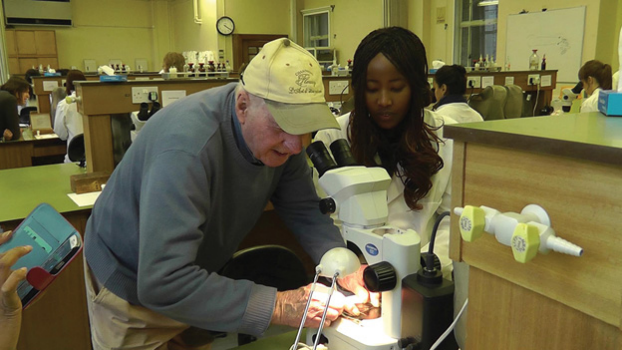
Tony Wilkes at the London School of Hygiene and Tropical Medicine. (Photo © the author, 2013)

In the lab. practical that follows the lecture, students are tasked with distinguishing between mosquitoes that have ovulated (parous) from those that have not (nulliparous)—a far simpler approach to assessing age. The Polovodova method, according to Mark Rowland, was “too discouraging to introduce this early on in an entomological career.” Tony makes his way around the laboratory, looking into various microscopes and, in some cases, helping students to better display their dissections. He becomes animated when he examines the work of a Kenyan entomologist whose dissection is particularly clear. Tony asks her whether or not she did the dissection fast or slow. She responds, “I need to do it quickly.” He smiles and nods, confirming the need for confidence and fluid motion: “you hesitate and you've lost the sample.” He picks up the dissection needles and starts to tease the ovary apart [Figure 10].

As Tony places his fingers on the slide, time redoubles. In the background, a VHS from the 1980s describing the more advanced Detinova technique is playing, detailing instructions over the ambient tones of a synthesizer that no one in the class is following. The Detinova method articulates the conditions of political and public health possibility at a particular moment in history, a moment that has long since elapsed. And yet the episodes explored in this article suggest that amongst the broader networks of references the microphotograph remains radically open to a re-visioning of global health futures and scientific pasts, and to the silenced histories of colonial labor and domestic lives, as a resource for future inquiry.

## Notes

Gillies had translated the articles during six months leave from Amani in 1957, having written to the Lenin Library to receive a copy of the paper in *Meditsinskaya Parazitologica i Parazitarniye Bolyezni* (Medical Parasitology and Parasitic Diseases) [Gillies 1958].

Mosquitoes can only transmit malaria parasites when at least ten days old. As temperature and humidity can affect egg development, there is some debate about the association between physiological age (the number of ovulations) and chronological age (the number of days lived). However, in tropical climates the length of the period of digestion and of egg maturation is more or less constant at 48 hours [Muirhead-Thomson 1951].

The experiment, conducted in Pare and Taveta districts in North-East Tanzania and South-West Kenya, was the brainchild of Bagster-Wilson, the Director of Amani Station, who took a hard line on the potentially deleterious effects of a large-scale spray campaign.

During the 19th and first quarter of the 20th century it was the most important endemic disease in Russia, at its height killing over 60,000 people per year.

If Gillies found himself cast in the role of a Western sceptic, he scripts Detinova as a dogmatic disciple of Soviet propaganda. “She lived for her work,” Gillies writes. “When I remarked how important it was for young people to visit each others’ countries she agreed, but insisted that they should go in groups. The unspoken fear of contamination by ideas from the outside world was only too apparent” [2000: 212].

A noted Marxist and communist sympathizer, as representatives from the United States were quick to point out.

The exhibit on the “American war crime of germ warfare” that toured major Chinese cities during the spring of 1952 including enlarged magnifications of insect heads (not unlike Robert Hooke's), highlights of the affective power and political potential of entomological imagery to tap into memories of the Japanese occupation, foment national sentiment and mobilize the population to contribute to public works and hygiene projects [Rogaski 2002: 385; Russell 1996].

In the transcript of the 19th World Health Assembly, Norgorodcev continues that the only way to rectify the situation was to follow the model of the Detinova mission, assisting the “sending of experts to countries, and particularly to the African countries … [by] the setting up of a commission of leading specialists from ten to fifteen countries, who would make a critical appraisal of the malaria eradication programme in the world over the last ten years” [WHO 1966: 246].

As Gillies notes, in the years following Independence, “In Amani little changed … one politically active member of the African laboratory, seen by us as a trouble maker, left and went into politics, but other than that scientific work progressed” [2000: 237].

Gillies seems troubled by the question of relevance: “What did it all add up to, the searches in the libraries of London, the importing of Detinova with her esoteric skills, first to London and then to embark on her grueling tour of Africa, and finally Tony's dedicated 18 months’ dissections at Amani? There is no simple answer to this question. Most biologists would agree that to possesses such a detailed picture of the life expectancy of any important insect could only be of the greatest interest. The dividends in practical terms were less impressive. No breakthrough in the control of malaria resulted and no claim could be made to have added to human happiness in the countries where we had worked” [2000: 225].
